# J-shape relationship between normal fasting plasma glucose and risk of type 2 diabetes in the general population: results from two cohort studies

**DOI:** 10.1186/s12967-023-04006-9

**Published:** 2023-03-05

**Authors:** Linfeng He, Wenbin Zheng, Zeyu Li, Lu Chen, Wen Kong, Tianshu Zeng

**Affiliations:** 1 Department of Endocrinology, Union Hospital, Tongji Medical College, Huazhong University of Science and Technology, Wuhan, Hubei China; 2 Hubei Provincial Clinical Research Center for Diabetes and Metabolic Disorders, Huazhong University of Science and Technology, Wuhan, Hubei China; 3 Hubei Key Laboratory of Metabolic Abnormalities and Vascular Aging, Huazhong University of Science and Technology, Wuhan, Hubei China

## Abstract

**Background:**

Previous studies have reported that high fasting plasma glucose (FPG), even that within the normal range, is associated with the risk of type 2 diabetes (T2D). Nevertheless, these findings are limited to specific populations. Thus, studies in the general population are imperative.

**Methods:**

This study included two cohorts comprising 204 640 individuals who underwent physical examinations at the Rich Healthcare Group present at 32 locations in 11 cities of China from 2010 to 2016 and 15 464 individuals who underwent physical tests at the Murakami Memorial Hospital in Japan. Cox regression, restricted cubic spline (RCS), Kaplan–Meier (KM) curves, and subgroup analysis were used to determine the relationship between FPG and T2D. Receiver operating characteristic (ROC) curves were used to evaluate the predictive power of FPG for T2D.

**Results:**

The mean age of the 220 104 participants (204 640 Chinese and 15 464 Japanese participants) was 41.8 years (41.7 years for the Chinese and 43.7 years for the Japanese participants). During follow-up, 2611 individuals developed T2D (2238 Chinese and 373 Japanese participants). The RCS demonstrated a J-shaped relationship between FPG and T2D risk, with inflexion points of 4.5 and 5.2 for the Chinese and Japanese populations, respectively. Multivariate-adjusted hazard ratio (HR) was 7.75 for FPG and T2D risk after the inflexion point (7.3 for Chinese and 21.13 for Japanese participants).

**Conclusions:**

In general Chinese and Japanese populations, the normal baseline FPG range showed a J-shaped relationship with the risk of T2D. Baseline FPG levels help identify individuals at high risk of T2D and may enable early primary prevention to improve their outcomes.

**Supplementary Information:**

The online version contains supplementary material available at 10.1186/s12967-023-04006-9.

## Introduction

The prevalence of T2D has rapidly increased over the past few decades. The harmful effects and increased healthcare costs associated with it are a growing public concern [1, 2]. According to the International Diabetes Federation, there were approximately 537 million cases of diabetes worldwide in 2019 [1]. Such individuals are at risk of developing associated complications, including heart disease, stroke, retinopathy, peripheral vascular disease, and kidney disease [3]. The health consequences and economic burden of the diabetes epidemic are enormous, with annual spending already exceeding USD 63.7 billion [1]. However, the current therapeutic strategies for diabetes can only partially prevent complications [4]. Therefore, the primary goals are prediction, prevention, and personalised treatment [5, 6].

Fasting plasma glucose (FPG) is closely related to diabetes, not only as an indicator of pancreatic function and insulin resistance [7] but also as a diagnostic criterion. The normal range for FPG was set at 3.9–6.1 mmol/L by the American Diabetes Association (ADA) in 1997 [8] and by the World Health Organization (WHO) in 1999 [4]. This was subsequently revised to 5.6 mmol/L by the ADA in 2003 [9]; however, the WHO standard remained unchanged [10]. Despite lowering the values of normal FPG range, they may not be effective in preventing T2D. Previous studies revealed that individuals with higher FPG, even those with levels in the normal range, are more likely to develop T2D [11–21]. Nevertheless, previous studies have focused on specific populations (young men, young adults, adults aged ≥ 30 years, healthy workers, middle-aged, and older people aged > 40 years, and nonobese people) [11–20] or a definite fasting glucose range (5–5.5 mmol/L) [21]. To our knowledge, there is a lack of studies in the general adult population (age ≥ 18 years) are lacking. In addition, the characteristics of T2D are inconsistent across ethnic populations [22].

Therefore, we analysed the relationship between normal FPG and T2D in a general adult population. Considering East Asians have a common ethnicity, they have similar diabetes characteristics and diagnostic criteria [23]. Therefore, both Chinese and Japanese cohorts were included in the analysis together.

## Methods

### Study populations and data collection

**Fig. 1:**
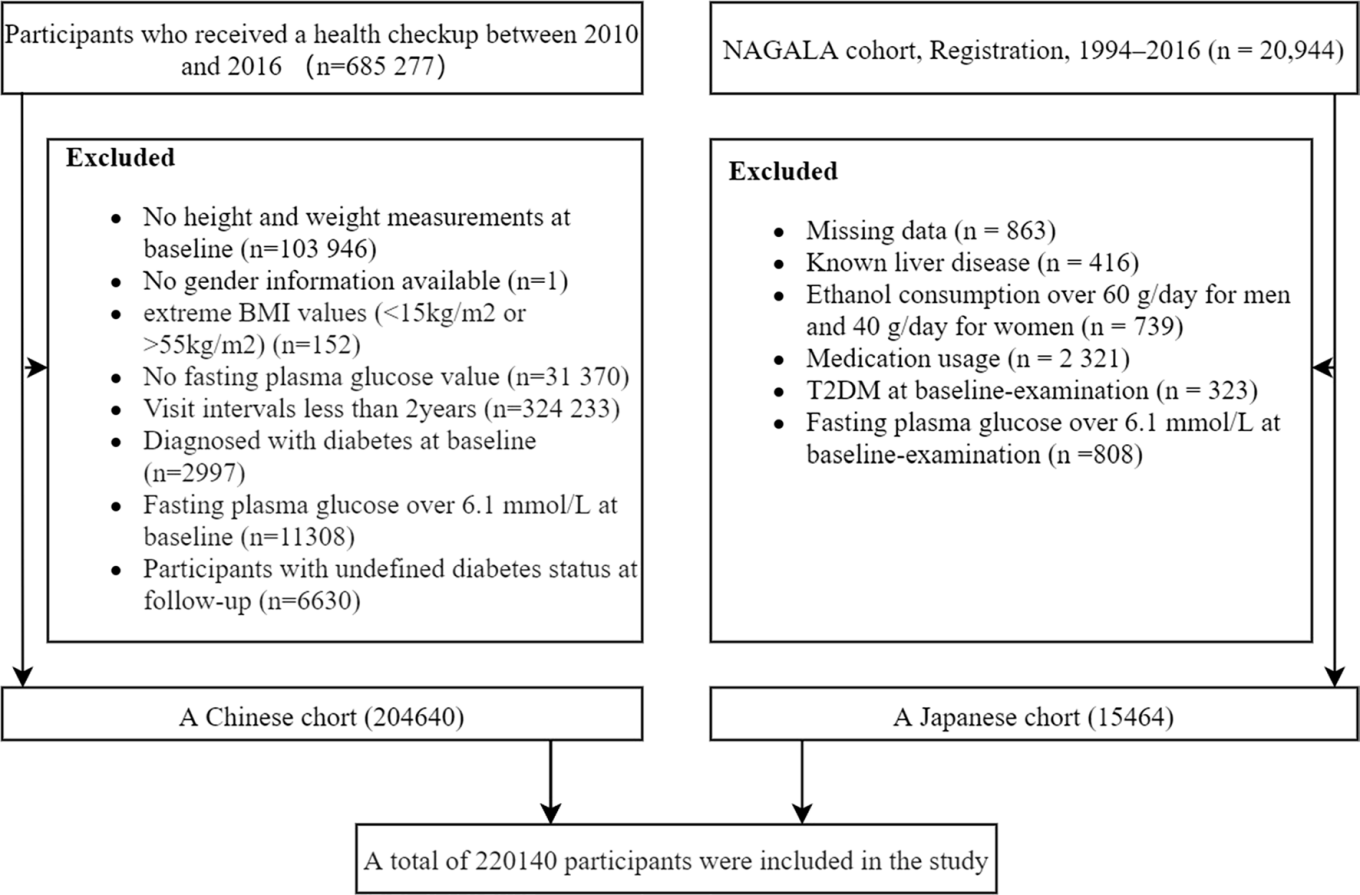
Flow chart for inclusion and exclusion of study participants

This study included the following two cohorts: (1) 685 277 individuals who were examined by the Rich Healthcare Group examined at 32 locations in 11 cities of China from 2010 to 2016 [24] and (2) the NAGALA (NAfld in the Gifu Area, Longitudinal Analysis) cohort from 1994 to 2016 [25]. Figure 1 illustrates the inclusion and exclusion criteria of the study.

FPG, serum triglyceride (TG), total cholesterol (TC), and alanine aminotransferase (ALT) levels were measured using an automated analyser after > 8 h of fasting. Questionnaires were used to record age, sex, height, weight, smoking, and alcohol history. Physicians measured blood pressure of the participants after 20 min of quiet rest.

### Definitions and follow-up

Hypertension was defined as systolic blood pressure (SBP) ≥ 140 mmHg or diastolic blood pressure (DBP) ≥ 90 mmHg. The diagnostic criteria for T2D were FPG ≥ 7.0 mmol/L, HbA1c of at least 6.5%, or self-reported T2D. Annual health checks were regarded as follow-up visits.

### Statistical analysis

First, all continuous data were tested for normality using Kolmogorov–Smirnov tests. For normally distributed data, analysis of variance (ANOVA) tests were used; for others, Kruskal–Wallis nonparametric tests were used to determine differences between the groups. Chi-square tests were used for comparing count data. Second, we conducted receiver operating characteristic (ROC) analysis to assess the prediction accuracy of FPG for T2D onset. Third, we performed Cox regression analysis to examine the linear relationship between FPG and new-onset T2D as well as restricted cubic spline (RCS) analysis to explore the potential nonlinear relationship. Fourth, the relationship between FPG and cumulative T2D incidence diabetes was analysed using Kaplan‒Meier (KM) curves. Finally, subgroup analysis was used to test the sensitivity analysis. All statistical analyses were conducted using R packages (http://www.r-project.org, R Foundation for Statistical Computing, Vienna, Austria) and EmpowerStats packages (www.empowerstats.com, X and Y Solutions, Inc., Boston MA, USA). Statistical significance was determined by *P* (two-sided) values below 0.05.

## Results

### Characteristics of the study population

**Fig. 2:**
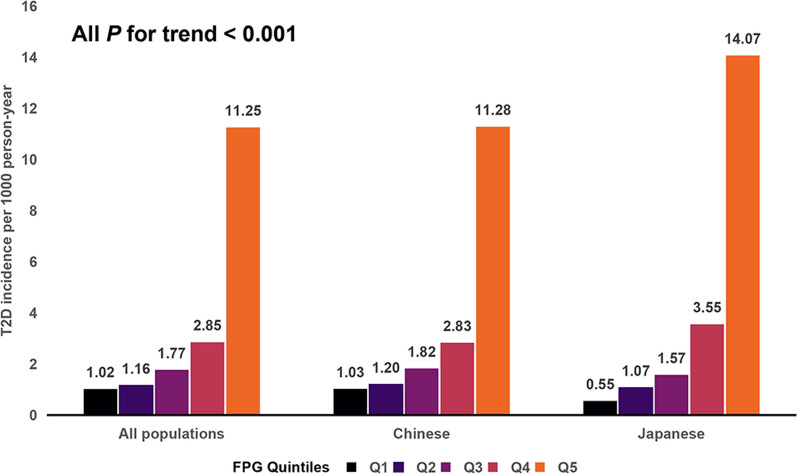
Incidence of T2D in different populations. **A**. All populations, **B**. Chinese, **C**. Japanese

A total of 220 104 people were included in this survey. The mean age was 41.8 years; 45.7% were men and 54.3% were women. Among them, 93% were Chinese and 7%, Japanese. The Chinese population were followed up for a mean period of 3.13 years, and 2238 people developed T2D during this time. The Japanese population were followed up for an average period of 6.05 years, and 373 people developed T2D during this follow-up time. The baseline characteristics of the study population are presented according to FPG quintiles (Table 1). FPG was significantly different from all baseline indicators (all *P* < 0.001). The Q5 group demonstrated the highest age, body mass index (BMI), SBP, DBP, TC, TG, and ALT, as well as the most significant number of women, smokers and drinkers, and persons with hypertension than the Q1–Q4 group (Table 1, Additional file 1: Tables S1, S2). Additionally, the Q5 group had the highest T2D incidence, with 11.28 cases per 1000 person-years among the Chinese population and 14.07 cases per 1000 person-years among the Japanese population (Fig. 2).

**Table 1:** Baseline characteristics of the all population according to FPG quintiles

	Total	Q1(≤ 4.44)	Q2(4.44–4.78)	Q3(4.78–5.04)	Q4(5.04–5.35)	Q5(≥ 5.35)	*P*-value
*N*	220104	44,021	44,021	44,020	444,021	44,021	
Age (years)	41.8 ± 12.2	39.5 ± 11.0	40.3 ± 11.7	41.0 ± 11.9	42.5 ± 12.3	45.9 ± 13.1	** < 0.001**
Sex							** < 0.001**
Women	100647 (45.7)	22,647 (51.4)	22,565 (51.3)	20,917 (47.5)	18,787 (42.7)	15,731 (35.7)	
Men	119457 (54.3)	21,374 (48.6)	21,456 (48.7)	23,103 (52.5)	25,234 (57.3)	28,290 (64.3)	
Country							** < 0.001**
Chinese	204640 (93.0)	43,468 (98.7)	41,540 (94.4)	41,149 (93.5)	39,436 (89.6)	39,047 (88.7)	
Japanese	15464 ( 7.0)	553 (1.3)	2481 (5.6)	2871 (6.5)	4585 (10.4)	4974 (11.3)	
BMI (kg/m^2^)	23.1 ± 3.3	22.4 ± 3.2	22.6 ± 3.2	22.9 ± 3.3	23.3 ± 3.3	24.1 ± 3.3	** < 0.001**
SBP (mmHg)	118.3 ± 16.1	114.8 ± 15.1	116.2 ± 15.5	117.6 ± 15.7	119.6 ± 16.0	123.6 ± 16.7	** < 0.001**
DBP (mmHg)	73.8 ± 10.7	72.5 ± 10.5	72.4 ± 10.5	73.2 ± 10.6	74.3 ± 10.6	76.6 ± 10.9	** < 0.001**
FPG (mmol/L)	4.9 ± 0.5	4.1 ± 0.3	4.6 ± 0.1	4.9 ± 0.1	5.2 ± 0.1	5.6 ± 0.2	** < 0.001**
TC (mmol/L)	4.7 ± 0.9	4.6 ± 0.9	4.6 ± 0.9	4.7 ± 0.9	4.8 ± 0.9	4.9 ± 0.9	** < 0.001**
TG (mmol/L)	1.0 (0.7, 1.5)	1.0 (0.7, 1.4)	1.0 (0.7, 1.4)	1.0 (0.7, 1.5)	1.1 (0.7, 1.6)	1.2 (0.8, 1.8)	** < 0.001**
ALT (U/L)	18.0 (12.9, 26.8)	16.7 (12.0, 25.0)	16.9 (12.0, 24.9)	17.3 (12.6, 26.0)	18.0 (13.0, 27.0)	20.0 (14.4, 30.4)	
Hypertension							** < 0.001**
No	192301 (87.4)	40,063 (91)	39,697 (90.2)	38,987 (88.6)	38,104 (86.6)	35,450 (80.5)	
Yes	27,803 (12.6)	3958 (9)	4324 (9.8)	5033 (11.4)	5917 (13.4)	8571 (19.5)	
Smoking							** < 0.001**
No	55,798 (25.4)	9683 (22)	10,310 (23.4)	10,860 (24.7)	12,388 (28.1)	12,557 (28.5)	
Yes	17,451 ( 7.9)	2618 (5.9)	2713 (6.2)	3137 (7.1)	3892 (8.8)	5091 (11.6)	
Unknown	146855 (66.7)	31,720 (72.1)	30,998 (70.4)	30,023 (68.2)	27,741 (63)	26,373 (59.9)	
Drinking							** < 0.001**
No	57,046 (25.9)	10,829 (24.6)	10,595 (24.1)	11,085 (25.2)	12,133 (27.6)	12,404 (28.2)	
Yes	16,203 ( 7.4)	1472 (3.3)	2428 (5.5)	2912 (6.6)	4147 (9.4)	5244 (11.9)	
Unknown	146855 (66.7)	31,720 (72.1)	30,998 (70.4)	30,023 (68.2)	27,741 (63)	26,373 (59.9)	

Measures are expressed as mean ± SD and median (IQR), and counts are expressed as n (%)

Bold values indicate the statistically significant *P* values

Continuous variables were compared between groups using ANOVA and Kruskal–Wallis analysis, with the chi-square test used for count data

*BMI* body mass index, *SBP* systolic blood pressure, *DBP* diastolic blood pressure, *FPG* fasting plasma glucose, *TC* total cholesterol, *TG* triglyceride, *ALT* alanine aminotransferase

### Relationship between baseline characteristics and T2D

To compare the role of FPG and other baseline parameters in predicting T2D, univariate Cox proportional hazards analysis was performed. In all populations, every baseline parameter (age, sex, BMI, SBP, DBP, FPG, TC, TG, ALT, smoking), except drinking were positively associated with the risk of developing T2D (all *P* < 0.001). The Chinese population demonstrated a consistent relationship, i.e, all parameters except drinking (*P* = 0.304) were positively correlated with T2D incidence. In the Japanese population, all parameters except smoking (*P* = 0.126) were positively correlated with the T2D incidence. Additional file 1: Table S3 shows the results of all univariate Cox regressions.

### Association between FPG and T2D

Higher FPG levels were associated with an increased risk of developing T2D in the fully model (Table 2). Multivariate-adjusted HRs (95% confidence interval [CI]) of the relationship between FPG and T2D in all populations, Chinese and Japanese, were 5.8 (5.31, 6.33), 5.5 (5.02, 6.02), and 14.57 (10.48, 20.25), respectively. We further divided the population into five FPG quintile groups (Q1–Q5), which were also positively correlated with the T2D incidence (all *P* for trend < 0.001).

**Table 2:** cox regression analysis of FPG versus T2D

	Crude model		Minimally model		Fully model	
	HR (95%CI)	*P-*value	HR (95%CI)	*P-*value	HR (95%CI)	*P-*value
All populations						
FPG	9.28 (8.5, 10.13)	** < 0.001**	6.5 (5.95, 7.1)	** < 0.001**	5.8 (5.31, 6.33)	** < 0.001**
FPG Quintiles						
Q1	Reference		Reference		Reference	
Q2	1.24 (1, 1.54)	**0.049**	1.16 (0.94, 1.44)	0.175	1.22 (0.98, 1.52)	0.072
Q3	2.01 (1.65, 2.46)	** < 0.001**	1.77 (1.45, 2.17)	** < 0.001**	1.81 (1.48, 2.21)	** < 0.001**
Q4	3.17 (2.64, 3.82)	** < 0.001**	2.55 (2.12, 3.07)	** < 0.001**	2.79 (2.32, 3.37)	** < 0.001**
Q5	12.73 (10.8, 15)	** < 0.001**	8.42 (7.13, 9.95)	** < 0.001**	8.07 (6.82, 9.56)	** < 0.001**
*P* for trend	** < 0.001**		** < 0.001**		** < 0.001**	
Chinese						
FPG	9.73 (8.9, 10.64)	** < 0.001**	6.8 (6.21, 7.44)	** < 0.001**	5.5 (5.02, 6.02)	** < 0.001**
FPG Quintiles						
Q1	Reference		Reference		Reference	
Q2	1.4 (1.12, 1.76)	**0.003**	1.3 (1.04, 1.63)	**0.022**	1.23 (0.98, 1.55)	0.07
Q3	2.38 (1.93, 2.93)	** < 0.001**	2.08 (1.69, 2.56)	** < 0.001**	1.85 (1.5, 2.27)	** < 0.001**
Q4	4.1 (3.38, 4.98)	** < 0.001**	3.28 (2.7, 3.99)	** < 0.001**	2.83 (2.32, 3.43)	** < 0.001**
Q5	16.8 (14.16, 19.93)	** < 0.001**	10.89 (9.16, 12.94)	** < 0.001**	8.26 (6.93, 9.83)	** < 0.001**
* P* for trend	** < 0.001**		** < 0.001**		** < 0.001**	
FPG	25.38 (18.71, 34.42)	** < 0.001**	21.83 (15.9, 29.98)	** < 0.001**	14.57 (10.48, 20.25)	** < 0.001**
Japanese						
FPG Quintiles						
Q1	Reference		Reference		Reference	
Q2	1.98 (0.95, 4.13)	0.069	1.89 (0.91, 3.96)	0.09	1.84 (0.86, 3.91)	0.115
Q3	2.89 (1.45, 5.77)	**0.003**	2.67 (1.33, 5.37)	**0.006**	2.3 (1.12, 4.72)	**0.023**
Q4	6.53 (3.44, 12.37)	** < 0.001**	5.88 (3.07, 11.29)	** < 0.001**	4.55 (2.31, 8.95)	** < 0.001**
Q5	26.24 (14.34, 48)	** < 0.001**	22.08 (11.85, 41.15)	** < 0.001**	14.21 (7.4, 27.28)	** < 0.001**
*P* for trend	** < 0.001**		** < 0.001**		** < 0.001**	

Statistical analysis method used: cox regression analysis

Bold values indicate the statistically significant *P* values

*Crude model*: unadjusted; *Minimally model*: adjusted for age and sex; *Fully model*: adjusted for age, sex, BMI, SBP, DBP, TC, TG, ALT, smoking, and drinking. All populations additional adjusted for country

*T2D* type 2 diabetes, *FPG* fasting plasma glucose, *HR* hazard ratio, *CI* confidence interval

**Table 3:** Threshold effect analysis of FPG and incident diabetes by piece-wise cox regression

	HR (95% CI)	*P-*value	*P* for log likelihood ratio test
All populations			** < 0.001**
FPG < 4.5 mmol/L	1.09 (0.66, 1.79)	0.732	
FPG ≥ 4.5 mmol/L	7.75 (6.97, 8.62)	** < 0.001**	
Chinese			** < 0.001**
FPG < 4.5 mmol/L	1.09 (0.66, 1.79)	0.738	
FPG ≥ 4.5 mmol/L	7.3 (6.53, 8.17)	** < 0.001**	
Japanes**e**			**0.048**
FPG < 5.2 mmol/L	3.22 (0.77, 13.38)	0.109	
FPG ≥ 5.2 mmol/L	21.13 (13.17, 33.91)	** < 0.001**	

Statistical analysis method used: piece-wise cox regression analysis and likelihood ratio test

Bold values indicate the statistically significant *P* values.

Adjusted for age, sex, BMI, SBP, DBP, TC, TG, ALT, smoking, and drinking. All populations additional adjusted for country

*T2D* type 2 diabetes, *FPG* fasting plasma glucose, *HR* hazard ratio, *CI* confidence interval

**Fig. 3:**
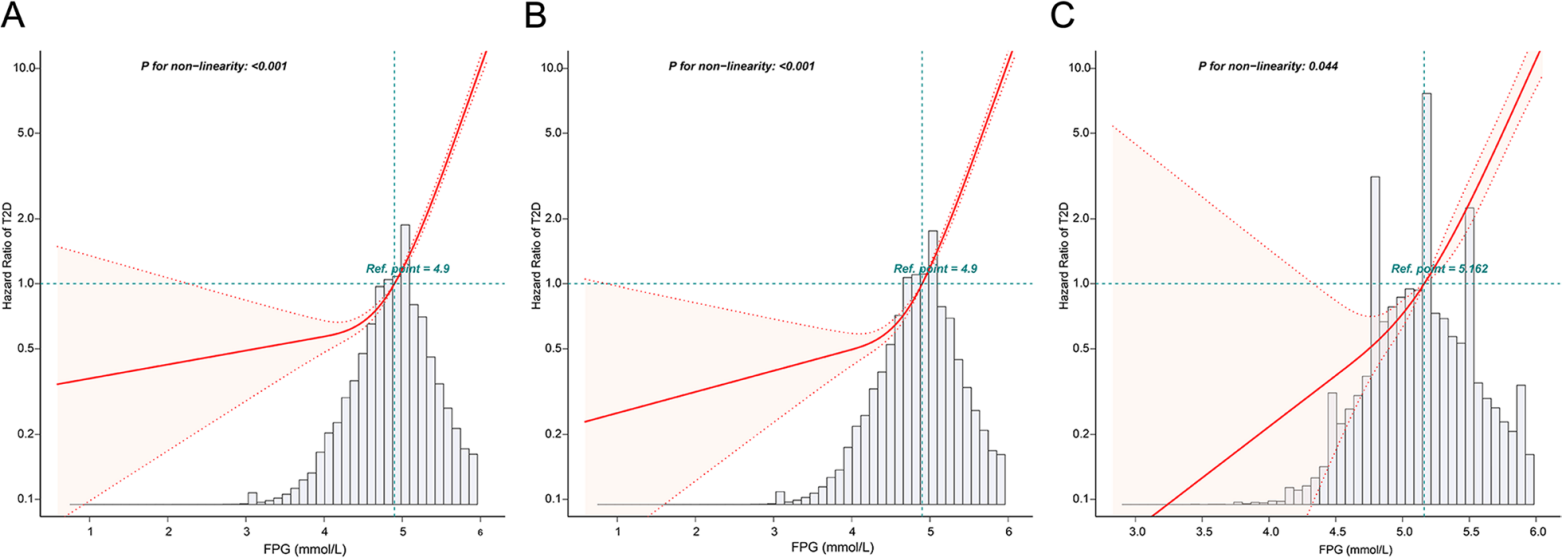
Five knots restricted cubic spline (RCS) plots of adjusted dose–response relationships for FPG and T2D risks in different populations, with density plots indicating the distribution of FPG. **A**. all populations, **B**. Chinese, **C**. Japanese. All models were adjusted for age, sex, BMI, SBP, DBP, TC, TG, ALT, smoking, and drinking. All populations were additionally adjusted for country

**Fig. 4:**
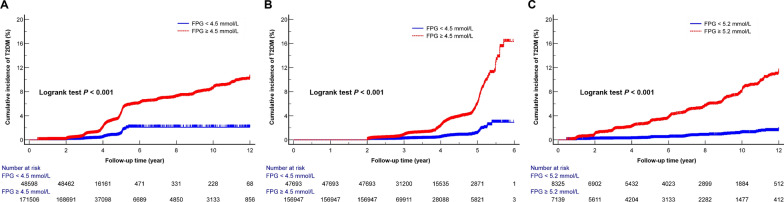
Kaplan–Meier plots of cumulative incidence of new-onset T2D in different populations grouped by RCS inflexion points, with log-rank test. **A**. All populations, **B**. Chinese, **C**. Japanese

We adjusted the variables of the fully model to further construct the RCS to depict the risk for FPG and T2D. Multivariate-adjusted RCS (Fig. 3) revealed a nonlinear relationship between FPG and new-onset T2D in all populations, Chinese, and Japanese. (all *P* for nonlinearity < 0.05). The inflexion points for the relationship between FPG and new-onset T2D in the Chinese and Japanese populations were 4.5 mmol/L and 5.2 mmol/L, respectively (Table 3). The risk of T2D increased rapidly with increasing FPG level beyond the inflexion point. On the right side of the inflexion point, the multivariate-adjusted HRs (95% CI) for all populations, Chinese and Japanese were 7.75 (6.97, 8.62), 7.3 (6.53, 8.17), and 21.13 (13.17, 33.91) respectively. We further divided the population into two groups based on the FPG inflexion points. The KM curves (Fig. 4) which were used to visualise the changes in T2D incidence between the two groups over the follow-up period showed a slow increase in T2D incidence in the groups to the left of the inflexion point. (all log-rank test *P* < 0.001).

### Predictive efficacy of FPG for new-onset T2D

The areas under the ROC curves (AUCs) were used to evaluate the effectiveness of FPG in predicting T2D risk. The AUCs of the FPG and T2D relationships were 0.772, 0.762, and 0.805 for all populations, Chinese, and Japanese, respectively, indicating good predictive performance (Additional file 1: Table S4, Fig. S1).

### Subgroup analysis of FPG and T2D

**Fig. 5:**
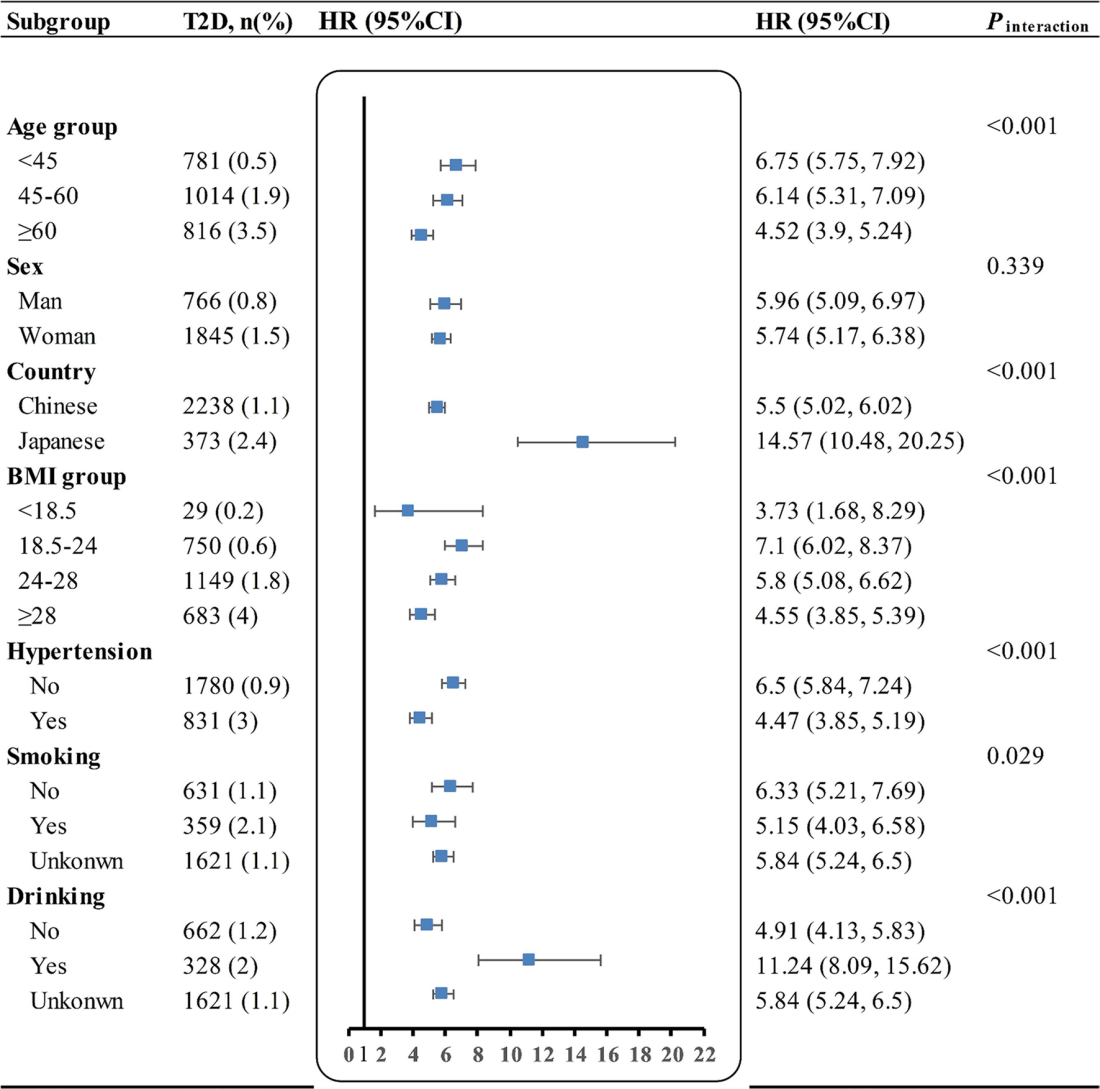
Forest plots of the relationship between FPG and new-onset T2D in different subgroups. Except for its stratification variables, all models adjusted for age, sex, country, BMI, SBP, DBP, TC, TG, ALT, smoking, and drinking

As a sensitivity analysis, we examined whether age, sex, country, BMI, hypertension, smoking, and drinking modified the relationship between the FPG and T2D. FPG was consistently positively associated with new-onset T2D in people stratified by age, sex, country, BMI, hypertension, smoking, and drinking. The multivariate-adjusted HRs (95% CI) are presented as a forest plot (Fig. 5). However, the relationship between FPG and T2D was correlated with age, BMI, hypertension, smoking, and drinking (all *P*_interaction_ < 0.05). In the Chinese and Japanese populations, a stronger correlation was observed between FPG and T2D in the following groups of people: age < 45 years, BMI (18.5–24 kg/m^2^), no hypertension, no smoking, and no drinking (Additional file 1: Table S5).

## Discussion

In this study, we observed a nonlinear relationship between the normal FPG range (3.9–6.1 mmol/L) and risk of future T2D occurrence in an East Asian population. The risk of T2D increased significantly when FPG exceeded 4.5 mmol/L and 5.2 mmol/L in the Chinese and Japanese populations, respectively. Overall, the rise in T2D risk in these the East Asian populations was insignificant or sluggish until FPG level reached 4.5 mmol/L.

Previous studies involving various populations exhibited similar results [11–21]. Tirosh et al. [19] reported an increased risk of T2D when FPG rose 87 mg/dL (4.83 mmol/L) in a group of 13 163 men aged 26–45 years tested at the Israeli Ministry of Defense Medical Examination Centre. Nichols et al. [17] determined that multivariate-adjusted HRs for FPG 90–94 mg/dL (5–5.2 mmol/L) and 95–99 mg/dL (5.3–5.5 mmol/dL) were 1.49 and 2.33, respectively in a US population comprising persons aged > 40 years. The difference between these HRs and those for FPG below 85 mg/dL (4.72 mmol/L), was statistically significant. A study involving an Italian population produced similar results [11]. To the best of our knowledge, only two such studies involving Chinese populations have been reported. In one, ROC curves were used to determine optimal FPG cut-off values for T2D prediction in 18 287 people aged ≥ 60 years in Taiwan, China (5.17 mmol/L and 5.11 mmol/L for men and women, respectively [12]. The other was the West China Hospital study involving a nonobese population (BMI < 25 kg/m2) adjusted for age, sex, family history of diabetes, waist circumference, BMI, SBP, and TG [20]. The results indicated a significantly increased risk of T2D in those with FPG > 4.3 mmol/L, which was similar to the cut-off point of 4.5 mmol/L in our study; However, the sample size was small (450 cases). Previous studies involving Japanese populations showed that a nonlinear relationship exists between FPG and T2D risk in people aged > 40 years [16, 18], male workers aged 40 years [15], and Japanese Americans aged 34–75 years [13]. However, the relationship between FPG 90–99 mg/dL (5–5.5 mmol/L) and T2D is controversial. Munekawa et al. [21] suggested that this could be attributed to distinct ages or populations. Consequently, in 2021, they confirmed that FPG 90–99 mg/dL (5–5.5 mmol/L) is associated with an increased risk of T2D using adult physical examination data from the Matsushita cohort in Japan. We found that the cut-off point beyond which the risk of T2D rapidly increased in the Japanese population was 5.2 mmol/L.

Previous epidemiological studies [26, 27] demonstrated that the incidence of diabetes progressively increases after the age of 40 years, suggesting the necessity of further research in younger populations. Therefore, we included people aged ≥ 18 years in our study and performed a stratified analysis to further elucidate the relationship between normal FPG range and risk of T2D. We found a stronger correlation between FPG and T2D in the following groups of people: age < 45 years, BMI (18.5–24 kg/m^2^), no hypertension, no smoking, and no drinking. Previous studies showed that age [28], obesity [29], hypertension [30], and smoking [31] are risk factors for T2D development. Consequently, the association between FPG and T2D may be weakened by the presence of these factors; however, drinking enhanced it, which might have been because the study population comprised light to moderate drinkers. Previous studies have shown that drinking is a double-edged sword for T2D; light to moderate drinking improves insulin sensitivity, while heavy drinking inhibits gluconeogenesis [32]. Thus, the effect of FPG on T2D is amplified in the drinking population. Nevertheless, the specific mechanisms regarding regulation of FPG and T2D by the factors mentioned above are unclear and need to be further explored.

Currently, the pathogenesis of T2D is not fully understood. Studies in European and American populations have shown that insulin resistance triggers T2D and ultimately leads to islet β-cell failure [33]. However, Asians, including the Chinese and Japanese, tend to have lower insulin levels and mild insulin resistance at T2D onset [23, 34, 35]. Mitsui et al. [36] reported that people with 100–109 mg/dL (5.6–6.1 mmol/L) FPG exhibit defective insulin secretion and insulin resistance. Therefore, the significantly increased risk of T2D associated with FPG > 4.5 mmol/L in the Chinese population and FPG > 5.2 mmol/L in the Japanese population observed in this study might have been due to defective insulin secretion and insulin resistance. At the cellular level, these homeostatic dysregulations cause cellular stress and mitochondrial malfunction [37, 38]. Increased reactive oxygen species (ROS) generation, mitochondrial membrane depolarisation, and decreased adenosine triphosphate synthesis are symptoms of mitochondrial malfunction [39]. To some extent, mitochondrial dysfunction may precede the emergence of insulin resistance [40]. Mitochondrial dysfunction affects insulin endocytosis in a dose- and time-dependent manner, reducing the expression of insulin receptor signalling [41]. Antioxidants, such as resveratrol and quercetin, reduce oxidative stress by scavenging ROS produced because of mitochondrial dysfunction [42, 43], thus aiding T2D treatment. Antioxidants act at an earlier stage of T2D pathogenesis than traditional insulinotropic agents, insulin sensitisers, and sodium-glucose cotransport protein 2 inhibitors and can serve as a new strategy for T2D treatment, with associated mitochondria-targeted antioxidant drugs showing good tolerability. Nevertheless, evidence for glycaemic control remains limited [44]. This study also provides a theoretical basis for the early application of mitochondria-targeted antioxidant therapy in the normoglycaemic phase.

### Study strengths and limitations

The strength of this study is the large sample size; this is the largest study population (over 200,000 people) for any study concerning normal FPG ranges and T2D risk. Moreover, our study covers a broader age range (18–97 years). However, our study has certain limitations. For the diagnosis of T2D, we relied solely on FPG, glycosylated haemoglobin, and patient self-reporting; therefore, oral glucose tolerance test (OGTT) might have been overlooked. Furthermore, there might have been confusion related to type 1 diabetes (T1D), which requires insulin antibody tests that time-consuming and laborious for diagnosis. We also did not have access to the medical history records of our study population; therefore, the possibility that certain medications caused T2D cannot be excluded.

## Conclusions

This study comprising over 200 000 general East Asians showed that a J-shaped relationship exists between normal FPG range and T2D risk, with inflexion points of 4.5 mmol/L and 5.2 mmol/L for the Chinese and Japanese populations, respectively. These results are expected to draw clinicians' attention to the risk of diabetes in a population exhibiting healthy glycaemic index currently. FPG levels below 4.5 mmol/L significantly reduce the risk of progression to T2D. Clinical trial interventions are still required to further validate our results. Future research, regarding early primary prevention and management of populations with healthy glucose levels currently is necessary.

## Supplementary Information

**Additional file 1: Fig. S1.** ROC curve analysis of the relationship between FPG and T2D in different populations. **Table S1.** Baseline characteristics of the Chinese population according to FPG quintiles. **Table S2.** Baseline characteristics of the Japanese population according to FPG quintiles. **Table S3.** Univariate cox regression analysis of T2D. **Table S4.** Efficacy of FPG in predicting T2D in different populations. **Table S5.** Subgroup analysis of FPG and T2D risk in the Chinese and Japanese populations.

**Additional file 2:** STROBE-cohort checklist.

## Data Availability

The data used in this study can be obtained from the 'DATADRYAD' database (www.Datadryad.org).

## Abbreviations

FPG: Fasting plasma glucose
T2D: Type 2 diabetes
RCS: Restricted cubic spline
KM: Kaplan–Meier
ADA: American diabetes association
WHO: World Health Organization
TG: Triglyceride
TC: Total cholesterol
ALT: Alanine aminotransferase
BMI: Body mass index
SBP: Systolic blood pressure
DBP: Diastolic blood pressure
ANOVA: Analysis of variance
ROC: Receiver operating characteristic
HR: Hazard ratio
CI: Confidence interval
AUC: Area under the curve
