# Supplementary material for: J-shape relationship between normal fasting plasma glucose and risk of type 2 diabetes in the general population: results from two cohort studies
Source: J Transl Med. 2023 Mar 5;21:175. doi: 10.1186/s12967-023-04006-9 (PMC9985867; doi:10.1186/s12967-023-04006-9)
Supplement: Supplementary file 1 — Additional file 1: Fig. S1. ROC curve analysis of the relationship between FPG and T2D in different populations. Table S1. Baseline characteristics of the Chinese population according to FPG quintiles. Table S2. Baseline characteristics of the Japanese population according to FPG quintiles. Table S3. Univariate cox regression analysis of T2D. Table S4. Efficacy of FPG in predicting T2D in different populations. Table S5. Subgroup analysis of FPG and T2D risk in the Chinese and Japanese populations. [file 12967_2023_4006_MOESM1_ESM.docx]

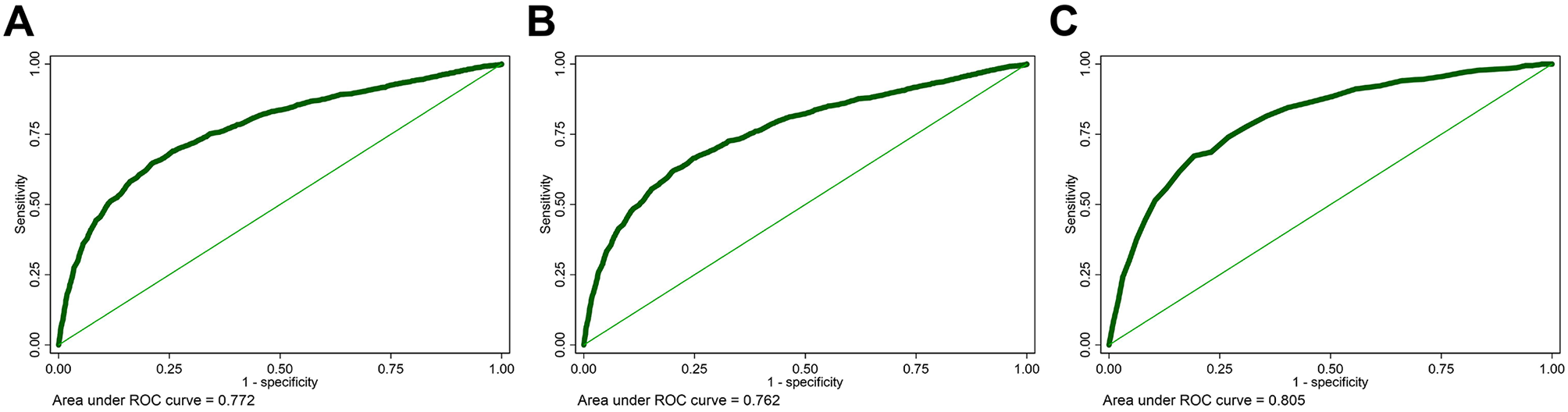


**Additional file 1: Fig. S1** ROC curve analysis of the relationship between FPG and T2D in different populations. A. All populations, B. Chinese, C. Japanese.

| Supplementary Table 1 Baseline characteristics of the Chinese population according to FPG quintiles | | | | | | | |
| --- | --- | --- | --- | --- | --- | --- | --- |
|  | **Total** | **Q1(≤4.41)** | **Q2(4.44-4.76)** | **Q3(4.76-5.01)** | **Q4(5.01-5.32)** | **Q5(≥5.32)** | **P-value** |
| *N* | 204640 | 40928 | 40928 | 40928 | 40928 | 40928 |  |
| Age (years) | 41.7 ± 12.4 | 39.5 ± 11.0 | 40.1 ± 11.8 | 40.9 ± 12.1 | 42.2 ± 12.6 | 45.9 ± 13.5 | **< 0.001** |
| **Sex** |  |  |  |  |  |  | **< 0.001** |
| Women | 93613 (45.7) | 20899 (51.1) | 20490 (50.1) | 19255 (47) | 17670 (43.2) | 15299 (37.4) |  |
| Men | 111027 (54.3) | 20029 (48.9) | 20438 (49.9) | 21673 (53) | 23258 (56.8) | 25629 (62.6) |  |
| BMI (kg/m^2^) | 23.2 ± 3.3 | 22.4 ± 3.2 | 22.7 ± 3.2 | 23.0 ± 3.3 | 23.4 ± 3.3 | 24.2 ± 3.3 | **< 0.001** |
| SBP (mmHg) | 118.6 ± 16.2 | 114.8 ± 15.1 | 116.6 ± 15.5 | 117.9 ± 15.7 | 120.0 ± 16.1 | 123.9 ± 16.8 | **< 0.001** |
| DBP (mmHg) | 74.0 ± 10.7 | 72.6 ± 10.5 | 72.7 ± 10.4 | 73.3 ± 10.6 | 74.5 ± 10.7 | 76.7 ± 10.9 | **< 0.001** |
| FPG (mmol/L) | 4.9 ± 0.6 | 4.1 ± 0.3 | 4.6 ± 0.1 | 4.9 ± 0.1 | 5.2 ± 0.1 | 5.6 ± 0.2 | **< 0.001** |
| TC (mmol/L) | 4.7 ± 0.9 | 4.6 ± 0.9 | 4.6 ± 0.9 | 4.7 ± 0.9 | 4.7 ± 0.9 | 4.9 ± 0.9 | **< 0.001** |
| TG (mmol/L) | 1.1 (0.7, 1.6) | 1.0 (0.7, 1.4) | 1.0 (0.7, 1.4) | 1.0 (0.7, 1.5) | 1.1 (0.8, 1.6) | 1.2 (0.9, 1.9) | **< 0.001** |
| ALT (U/L) | 18.0 (12.9, 27.0) | 16.8 (12.0, 25.1) | 17.0 (12.0, 25.0) | 17.6 (12.7, 26.2) | 18.0 (13.0, 27.7) | 20.1 (14.3, 30.9) | **< 0.001** |
| **Hypertension** |  |  |  |  |  |  | **< 0.001** |
| No | 177801 (86.9) | 37218 (90.9) | 36753 (89.8) | 36091 (88.2) | 35163 (85.9) | 32576 (79.6) |  |
| Yes | 26839 (13.1) | 3710 (9.1) | 4175 (10.2) | 4837 (11.8) | 5765 (14.1) | 8352 (20.4) |  |
| **Smoking** |  |  |  |  |  |  | **< 0.001** |
| No | 43993 (21.5) | 8625 (21.1) | 8078 (19.7) | 8470 (20.7) | 9242 (22.6) | 9578 (23.4) |  |
| Yes | 13792 ( 6.7) | 2385 (5.8) | 2346 (5.7) | 2595 (6.3) | 2853 (7) | 3613 (8.8) |  |
| Unkonwn | 146855 (71.8) | 29918 (73.1) | 30504 (74.5) | 29863 (73) | 28833 (70.4) | 27737 (67.8) |  |
| **Drinking** |  |  |  |  |  |  | **< 0.001** |
| No | 48015 (23.5) | 9774 (23.9) | 8719 (21.3) | 9119 (22.3) | 9890 (24.2) | 10513 (25.7) |  |
| Yes | 9770 ( 4.8) | 1236 (3) | 1705 (4.2) | 1946 (4.8) | 2205 (5.4) | 2678 (6.5) |  |
| Unkonwn | 146855 (71.8) | 29918 (73.1) | 30504 (74.5) | 29863 (73) | 28833 (70.4) | 27737 (67.8) |  |

Measures are expressed as mean ± SD and median (IQR), and counts are expressed as n (%);

Continuous variables were compared between groups using ANOVA and Kruskal-Wallis analysis, with the chi-square test used for count data.

BMI, body mass index; SBP, systolic blood pressure; DBP, diastolic blood pressure; FPG, fasting plasma glucose; TC, total cholesterol;

TG, triglyceride; ALT, alanine aminotransferase

| **Additional file 1**Table S2 Baseline characteristics of the Japanese population according to FPG quintiles | | | | | | | |
| --- | --- | --- | --- | --- | --- | --- | --- |
|  | **Total** | **Q1(≤4.82)** | **Q2(4.82-5.05)** | **Q3(5.05-5.27)** | **Q4(5.27-5.55)** | **Q5(>5.55)** | ***P*-value** |
| *N* | 15464 | 3093 | 3093 | 3092 | 3093 | 3093 |  |
| Age (years) | 43.7 ± 8.9 | 41.8 ± 8.5 | 42.8 ± 8.8 | 43.6 ± 8.8 | 44.3 ± 8.8 | 46.1 ± 9.0 | **< 0.001** |
| **Sex** |  |  |  |  |  |  | **< 0.001** |
| Women | 7034 (45.5) | 2386 (77.1) | 1881 (60.8) | 1291 (41.8) | 867 (28) | 609 (19.7) |  |
| Men | 8430 (54.5) | 707 (22.9) | 1212 (39.2) | 1801 (58.2) | 2226 (72) | 2484 (80.3) |  |
| BMI (kg/m^2^) | 22.1 ± 3.1 | 20.6 ± 2.6 | 21.4 ± 2.8 | 22.1 ± 3.0 | 22.8 ± 3.1 | 23.6 ± 3.2 | **< 0.001** |
| SBP (mmHg) | 114.5 ± 15.0 | 107.0 ± 13.2 | 111.1 ± 13.7 | 115.1 ± 14.4 | 117.7 ± 14.1 | 121.7 ± 14.9 | **< 0.001** |
| DBP (mmHg) | 71.6 ± 10.5 | 66.5 ± 9.4 | 69.2 ± 9.7 | 71.9 ± 10.0 | 73.6 ± 10.0 | 76.6 ± 10.4 | **< 0.001** |
| FPG (mmol/L) | 5.2 ± 0.4 | 4.6 ± 0.2 | 4.9 ± 0.1 | 5.1 ± 0.1 | 5.4 ± 0.1 | 5.7 ± 0.2 | **< 0.001** |
| TC (mmol/L) | 5.1 ± 0.9 | 4.9 ± 0.8 | 5.0 ± 0.9 | 5.1 ± 0.9 | 5.2 ± 0.8 | 5.4 ± 0.9 | **< 0.001** |
| TG (mmol/L) | 0.7 (0.5, 1.1) | 0.6 (0.4, 0.8) | 0.6 (0.4, 0.9) | 0.7 (0.5, 1.1) | 0.8 (0.6, 1.3) | 1.0 (0.7, 1.5) |  |
| ALT (U/L) | 17.0 (13.0, 23.0) | 14.0 (11.0, 18.0) | 15.0 (12.0, 20.0) | 17.0 (13.0, 23.0) | 18.0 (14.0, 25.0) | 21.0 (15.0, 29.0) |  |
| **Hypertension** |  |  |  |  |  |  | **< 0.001** |
| No | 14500 (93.8) | 3031 (98) | 2982 (96.4) | 2914 (94.2) | 2855 (92.3) | 2718 (87.9) |  |
| Yes | 964 ( 6.2) | 62 (2) | 111 (3.6) | 178 (5.8) | 238 (7.7) | 375 (12.1) |  |
| **Smoking** |  |  |  |  |  |  | **< 0.001** |
| No | 11805 (76.3) | 2694 (87.1) | 2533 (81.9) | 2370 (76.6) | 2194 (70.9) | 2014 (65.1) |  |
| Yes | 3659 (23.7) | 399 (12.9) | 560 (18.1) | 722 (23.4) | 899 (29.1) | 1079 (34.9) |  |
| Unkonwn | - | - | - | - | - | - |  |
| **Drinking** |  |  |  |  |  |  | **< 0.001** |
| No | 9031 (58.4) | 2282 (73.8) | 2081 (67.3) | 1742 (56.3) | 1565 (50.6) | 1361 (44) |  |
| Yes | 6433 (41.6) | 811 (26.2) | 1012 (32.7) | 1350 (43.7) | 1528 (49.4) | 1732 (56) |  |
| Unkonwn | - | - | - | - | - | - |  |

Measures are expressed as mean ± SD and median (IQR), and counts are expressed as n (%);

Continuous variables were compared between groups using ANOVA and Kruskal-Wallis analysis, with the chi-square test used for count data;

BMI, body mass index; SBP, systolic blood pressure; DBP, diastolic blood pressure; FPG, fasting plasma glucose; TC, total cholesterol;

TG, triglyceride; ALT, alanine aminotransferase

| **Additional file 1**Table S3 Univariate cox regression analysis of T2D | | | | | | | | |
| --- | --- | --- | --- | --- | --- | --- | --- | --- |
|  | **All poputions** | |  | **Chinese** | |  | **Japenese** | |
|  | **HR(95%CI)** | **P-value** |  | **HR(95%CI)** | **P-value** |  | **HR(95%CI)** | **P-value** |
| Age | 1.07 (1.06,1.07) | **< 0.001** |  | 1.06 (1.06,1.07) | **< 0.001** |  | 1.06 (1.04,1.07) | **< 0.001** |
| Sex |  | **< 0.001** |  |  | **< 0.001** |  |  | **< 0.001** |
| Woman | Reference |  |  | Reference |  |  | Reference |  |
| Man | 1.96 (1.8,2.13) |  |  | 1.89 (1.72,2.06) |  |  | 2.52 (1.98,3.21) |  |
| BMI | 1.25 (1.24,1.26) | **< 0.001** |  | 1.24 (1.23,1.26) | **< 0.001** |  | 1.24 (1.22,1.27) | **< 0.001** |
| SBP | 1.04 (1.04,1.04) | **< 0.001** |  | 1.04 (1.04,1.04) | **< 0.001** |  | 1.03 (1.03,1.04) | **< 0.001** |
| DBP | 1.05 (1.05,1.05) | **< 0.001** |  | 1.05 (1.04,1.05) | **< 0.001** |  | 1.05 (1.04,1.06) | **< 0.001** |
| FPG | 9.28 (8.5,10.13) | **< 0.001** |  | 9.73 (8.9,10.64) | **< 0.001** |  | 25.38 (18.71,34.42) | **< 0.001** |
| TC | 1.35 (1.3,1.4) | **< 0.001** |  | 1.4 (1.34,1.46) | **< 0.001** |  | 1.49 (1.34,1.66) | **< 0.001** |
| TG | 1.29 (1.28,1.31) | **< 0.001** |  | 1.27 (1.25,1.29) | **< 0.001** |  | 1.8 (1.68,1.92) | **< 0.001** |
| [AL](file:///D:\%25E5%25AE%2589%25E8%25A3%2585%25E7%25A8%258B%25E5%25BA%258F\Youdao\Dict\9.0.4.0\resultui\html\index.html#\javascript:;)T | 1.01 (1.00,1.01) | **< 0.001** |  | 1.01 (1.00,1.01) | **< 0.001** |  | 1.01 (1.01,1.01) | **< 0.001** |
| Smoking |  | **< 0.001** |  |  | **< 0.001** |  |  | 0.126 |
| No | Reference |  |  | Reference |  |  | Reference |  |
| Yes | 1.74 (1.52,1.98) |  |  | 2.15 (1.83,2.52) |  |  | 1.19 (0.95,1.49) |  |
| Unkonwn | 1.8 (1.63,1.99) |  |  | 1.34 (1.2,1.51) |  |  | - |  |
| Drinking |  | **< 0.001** |  |  | 0.304 |  |  | **< 0.001** |
| No | Reference |  |  | Reference |  |  | Reference |  |
| Yes | 1.07 (0.94,1.22) |  |  | 0.88 (0.71,1.1) |  |  | 2.17 (1.76,2.67) |  |
| Unkonwn | 1.56 (1.42,1.72) |  |  | 1.03 (0.93,1.14) |  |  | - |  |
| Statistical analysis method used: cox regression analysis.  T2D, type 2 diabetes; BMI, body mass index; SBP, systolic blood pressure; DBP, diastolic blood pressure; FPG, fasting plasma glucose; TC total cholesterol; TG, triglyceride; ALT, alanine aminotransferase; HR, hazard ratio; CI, confidence interval | | | | | | | | |

| **Additional file 1:** Table S4 Efficacy of FPG in predicting T2D in different populations | | | | |
| --- | --- | --- | --- | --- |
|  | **Cut-off value** | **AUC (95%CI)** | **Sensitivity (%)** | **Specificity (%)** |
| All populations | 5.3 | 0.772 (0.77, 0.774) | 64.84 | 78.7 |
| Chinese | 5.3 | 0.762 (0.76, 0.764) | 62.06 | 79.76 |
| Japanese | 5.5 | 0.805 (0.799, 0.811) | 67.29 | 80.79 |
| Statistical analysis method used: ROC curves analysis.  AUC, area under the ROC curve; CI, confidence interval | | | | |

| **Additional file 1:** Table S5 Subgroup analysis of FPG and T2D risk in the Chinese and Japanese populations | | | | | |
| --- | --- | --- | --- | --- | --- |
| Subgroup | **Chinese** | |  | **Japanese** | |
|  | **HR (95%CI)** | ***P*interaction** |  | **HR (95%CI)** | ***P*interaction** |
| Age group |  | **<0.001** |  |  | **0.02** |
| <45 | 6.19 (5.24, 7.31) |  |  | 20.9 (12.37, 35.3) |  |
| 45-60 | 5.82 (5, 6.77) |  |  | 12.15 (7.63, 19.35) |  |
| ≥60 | 4.51 (3.88, 5.24) |  |  | 7.06 (2.45~20.32) |  |
| Sex |  | 0.316 |  |  | 0.941 |
| Man | 5.63 (4.79, 6.62) |  |  | 13.11 (7.22, 23.81) |  |
| Woman | 5.44 (4.88, 6.06) |  |  | 14.6 (9.77, 21.82) |  |
| BMI group |  | **<0.001** |  |  | **0.037** |
| <18.5 | 4.14 (2.24, 7.65) |  |  | 16.9 (1.89, 151.45) |  |
| 18.5-24 | 6.21 (5.22, 7.37) |  |  | 22.97 (14.01, 37.66) |  |
| 24-28 | 5.7 (4.98, 6.53) |  |  | 11.33 (6.43, 19.96) |  |
| ≥28 | 4.47 (3.75, 5.32) |  |  | 9.31 (4.17, 20.78) |  |
| Hypertension |  | **<0.001** |  |  | 0.261 |
| No | 6.05 (5.41, 6.77) |  |  | 15.44 (10.86, 21.94) |  |
| Yes | 4.42 (3.8, 5.13) |  |  | 10.36 (3.86, 27.79) |  |
| Smoking |  | 0.05 |  |  | 0.974 |
| No | 5.4 (4.31, 6.76) |  |  | 12.1 (7.3, 20.06) |  |
| Yes | 4.13 (3.19, 5.34) |  |  | 15.69 (10.13, 24.31) |  |
| Unkonwn | 5.84 (5.24, 6.5) |  |  | - |  |
| Drinking |  | **0.032** |  |  | **0.033** |
| No | 4.52 (3.77, 5.42) |  |  | 11.69 (8.03, 17) |  |
| Yes | 8 (4.88, 13.1) |  |  | 29.24 (14.52, 58.89) |  |
| Unkonwn | 5.84 (5.24, 6.5) |  |  | - |  |

Statistical analysis method used: cox regression analysis and interaction tests;

Except for its stratification variables, all models adjusted for age, sex, country, BMI, SBP, DBP, TC, TG, ALT, smoking, and drinking.

T2D, type 2 diabetes; BMI, body mass index; FPG, fasting plasma glucose; HR, hazard ratio; CI, confidence interval
